# Structural aortic wall abnormalities following the Nikaidoh operation, which could be reversible and include a healing process

**DOI:** 10.21542/gcsp.2024.3

**Published:** 2024-01-03

**Authors:** Magdi H. Yacoub, Ahmed Afifi, Hatem Hosny, Ahmed Mahgoub, Mohamed Nagy, Sanida Vaz, Padmini Sarathchandra, Najma Latif

**Affiliations:** 1 Magdi Yacoub Institute, National Heart and lung Institute, Imperial College London, London, UK; 2 Cardiac surgery department, Aswan Heart Centre, Aswan, Egypt

## Abstract

The Nikaidoh operation continues to be used for patients with transposition of the great arteries, ventricular septal defect and left ventricular outflow tract obstruction. We recently reported structural and functional changes in the aortic root during the follow-up of a patient who underwent the Nikaidoh operation. These changes necessitated re-operation. The pathophysiology of these changes and their potential for reversibility have not yet been studied. In this communication, we describe the extensive structural changes in the aortic wall of the same patient.

## Introduction

The Nikaidoh operation^1,2^ shows excellent early results; however, long-term changes in the aortic wall remain undefined. We have recently reported the structure and function of the aortic and pulmonary outflows in a patient 12 years after the Nikaidoh operation^3^. Clinical aspects have been reported^3^ however its pathophysiology is unknown. Here, we show changes in the extracellular matrix and cellular components of the aortic wall, which include the healing process. These findings have important clinical implications.

**Figure 1.**
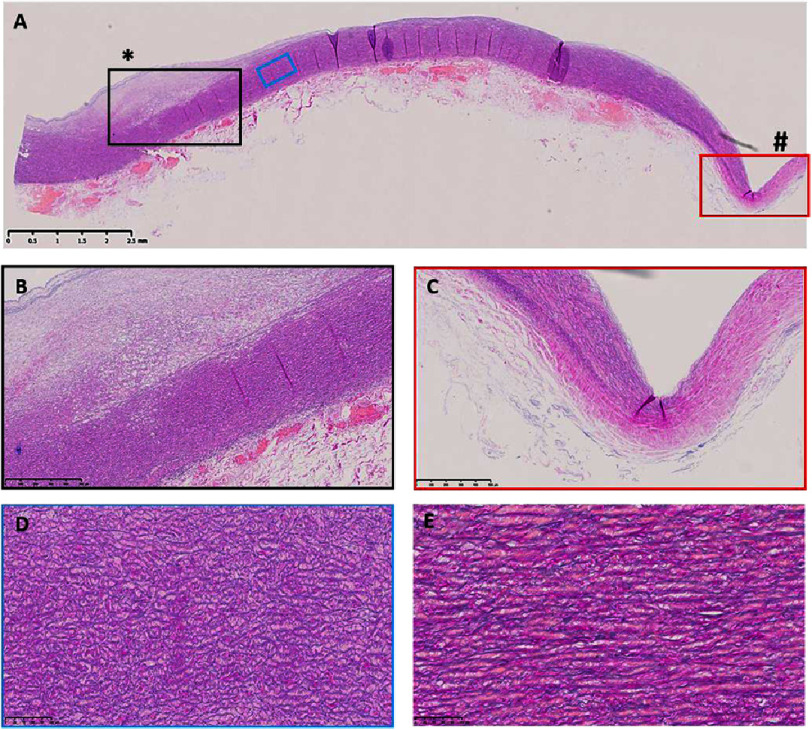
EVG staining of the Nikaidoh aortic wall. The wall has a bulge (*) between the internal elastic lamina and the media on the left (A, B). The other end of the wall is very thin and has no lamellae (#, C). The lamellae (blue box, D) are not as parallel as a control (E). Scale bars are on each panel.

**Figure 2.**
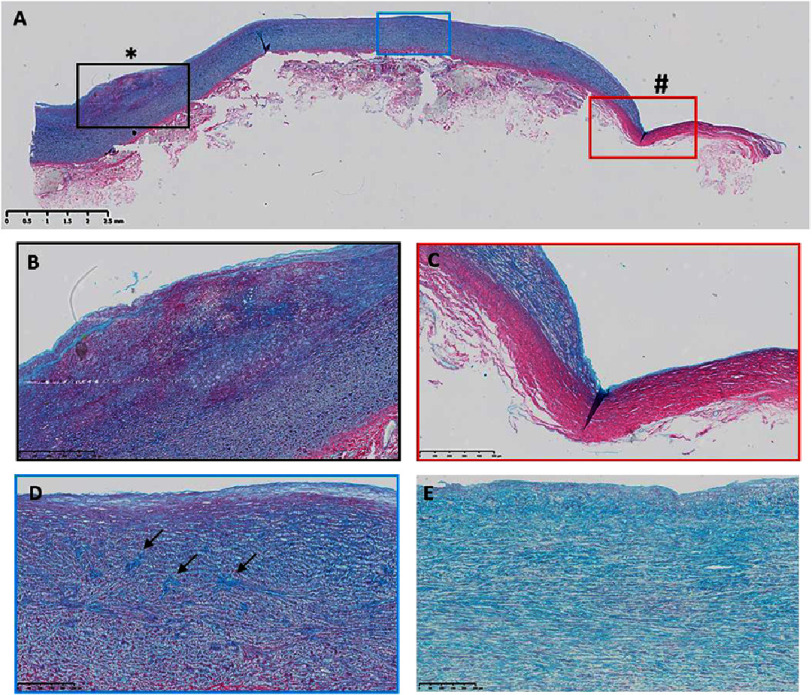
Alcian blue and Picrosirius red staining of the Nikaidoh aortic wall. The bulge (*) consists of glycosaminoglycans (blue) intermingled with collagen (pink) (A, B). The other end of the wall (#) is very thin and has no glycosaminoglycans but abundant collagen (A, C). The medial collagen (blue box, D) alignment is not as parallel as a control (E) and shows aggregation of gylcosaminoglycans (arrows). Scale bars are on each panel.

**Figure 3.**
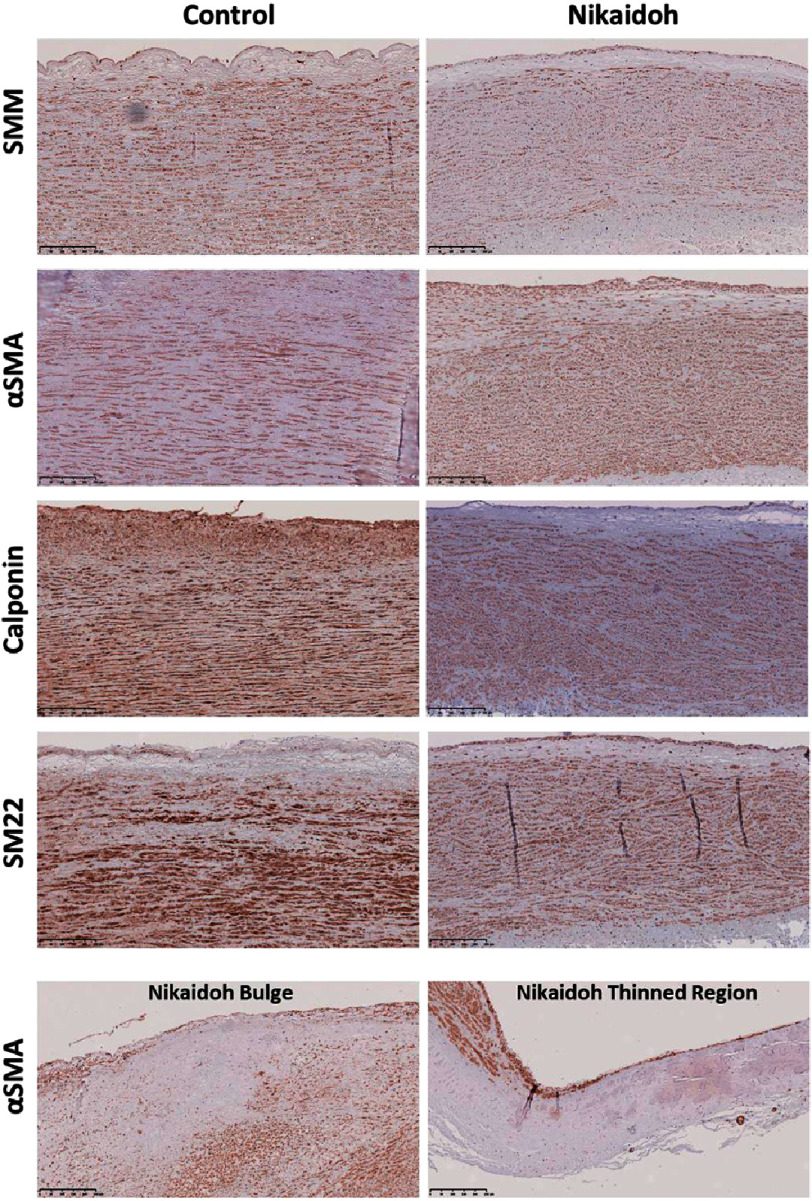
Immunostaining of the aortic wall with smooth muscle markers of a control and the Nikaidoh sample. Scale bars are on each panel.

**Figure 4.**
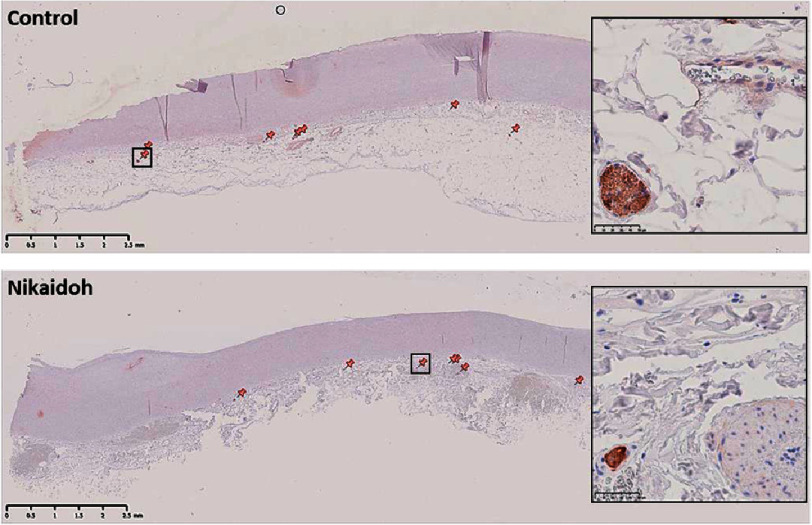
Distribution, number and size of nerve bundles in a control and the Nikaidoh aortic wall. Staining is with anti-neurofilament antibody. The insets show magnified images of the small region in each panel. Scale bars are on each panel.

**Figure 5.**
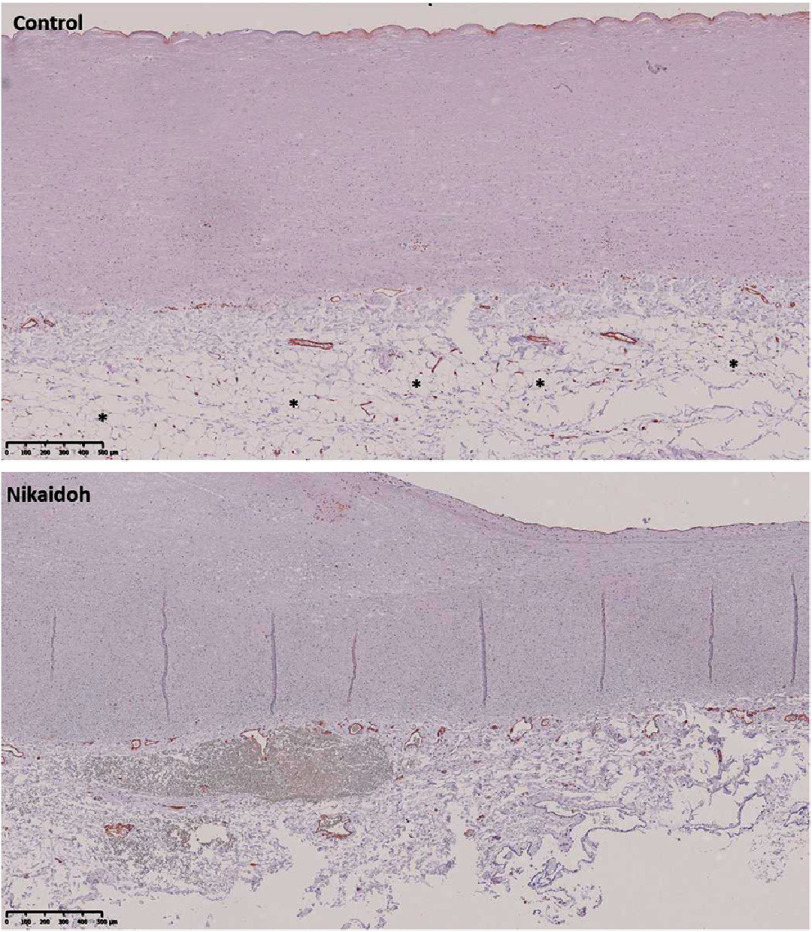
Staining of endothelial cells in a control and the Nikaidoh aortic wall. Staining is with an antibody against CD31. The lumen with the endothelial layer is at the top in each panel. * Indicates adipocytes. Scale bars are on each panel.

### Patient and methods

This patient was operated at the age of 2 years, and at age 14 years, showed severe dilatation of the non-coronary sinus of the aortic root, compressing the RVOT and pulmonary branches, resulting in RV dilatation^3^. We used three normal aortic wall specimens (mean age: 62.33 years, SD = 7.02; all males) to compare the changes observed in this Nikaidoh aortic wall sample. Ethical approval was obtained from the Aswan Heart Centre ethical board for the use of explanted aortic wall tissue and from the Royal Brompton Hospital for the use of control samples.

### Histological and immunological staining

Histological (elastic van Gieson, Alcian blue and Picrosirius red) and immunological staining procedures were as described before^4,5^. Anti-neurofilament antibody (Dako) was used at a dilution of 1/200 and anti-CD31 antibody (Abcam) at 1/300 dilution. Slides were scanned using a Nanozoomer (Hamamatsu, Japan).

## Results

The Nikaidoh aortic wall showed bulging at one end and thinning at the other end of the segment, which was explanted (Figure 1A). The bulging was between the internal elastic lamina and the media. This bulge contains very little elastin (Figure 1B). Similarly, the thinned area showed an almost total loss of elastin and lamellae (Figure 1C), and loss of the main contractile components. At high magnification, the lamellae in the aortic wall of the Nikaidoh sample showed a lack of parallel alignment (Figure 1D) compared to control samples (Figure 1E).

Further analysis showed that the bulge showed increased collagen and glycosaminoglycan (GAG) deposition (Figure 2A, B). The thinned-out region showed a complete loss of GAGs, which were replaced by fibrotic collagen (Figure 1C). The media showed increased levels of GAGs and collagen throughout the regions of aggregated GAG deposition (Figure 1D) compared to control samples (Figure 1E).

By analyzing the smooth muscle cellular component, we found that all the main smooth muscle contractile markers analyzed were reduced in expression (smooth muscle cell myosin (SMM), *α*-smooth muscle actin ( *α*SMA), calponin, and SM22) (Figure 3), with punctate expression compared to elongated cellular expression in the control samples. Furthermore, the bulge region showed sparse expression of smooth muscle markers, indicating a loss in the number of smooth muscle cells, and the thinned-out region was almost devoid of staining for smooth muscle markers (Figure 3). This area matched the same region devoid of lamellae (Figure 1C) and replaced with collagen (Figure 2C).

We analyzed the regulatory elements of the adventitia that influence the media and abundance of nerves and vessels. The mean thickness of the normal section (excluding the bulge and thinner region) of the media of the Nikaidoh sample was thinner than the mean thickness of the control aortic walls (0.78 mm, SD = 0.06 vs 1.69 mm, SD = 0.38) (Figures 4 and 5). The Nikaidoh sample had a similar number of nerve bundles (7.00 vs 6.33 respectively) but a smaller size of nerve bundles compared to controls (mean diameter, 39.26 μm, SD = 32.29 μm vs 67.60 μm, SD = 38.85 μm, respectively) when stained with anti-neurofilament antibody (Figure 4) in a similar length of the aortic wall. The nerve bundles were spread along the length of both the Nikaidoh and the control aortic wall samples. Surface endothelial cell and adventitial vessel staining were similar in the controls and the Nikaidoh sample. Interestingly, the Nikaidoh sample lacked adventitial adipocytes.

## Discussion

Here, we show, for the first time, changes in the structural composition of the aortic wall following the Nikaidoh operation, which includes regulatory elements in the adventitia as well as changes in the main extracellular matrix proteins. The organization and content of elastin and collagen were reduced with a loss of alignment of the lamellae. The cellular content also showed deterioration in that smooth muscle cell contractile proteins were reduced with loss of their elongated morphology. In addition, there was an attempt at healing evidenced by the presence of innervation and vascularisation, which could, perceivably, be used to reverse the injury to the aortic wall. The lack of adipose tissue in the outer adventitial wall could have major consequences for the homeostasis of the aortic wall, as adventitial adipose tissue regulates the structure and function of normal arterial walls through autocrine and paracrine mechanisms, including modulation of medial smooth muscle contractility and secretion of anti-inflammatory molecules^6,7^.

### What have we learned?

The original data presented in this manuscript defining the detailed complex pathophysiology of the Nikaidoh arterial wall could have major clinical implications for preventing or reversing these changes.
